# Aspirin Action in Endothelial Cells: Different Patterns of Response Between Chemokine CX3CL1/CX3CR1 and TNF-α/TNFR1 Signaling Pathways

**DOI:** 10.1007/s10557-015-6589-2

**Published:** 2015-05-12

**Authors:** Dariusz Szukiewicz, Malgorzata Wojciechowska, Anna Bilska, Aleksandra Stangret, Grzegorz Szewczyk, Tarun Kumar Mittal, Mateusz Watroba, Jan Kochanowski

**Affiliations:** Department of General & Experimental Pathology with Centre for Preclinical Research and Technology (CEPT), Medical University of Warsaw, ul. Pawinskiego 3C, 02-106 Warsaw, Poland; Department of Obstetrics & Gynecology, Second Faculty of Medicine, Medical University of Warsaw, ul. Kondratowicza 8, 03-242 Warsaw, Poland; Department of Neurology, Second Faculty of Medicine, Medical University of Warsaw, ul. Ceglowska 80, 01-809 Warsaw, Poland

**Keywords:** Aspirin, Endothelial cells, Fractalkine, CX3CR1, TNF-α, TNFR1

## Abstract

**Purpose:**

TNF-α induces fractalkine (CX3CL1) and its receptor CX3CR1 in endothelial cells through NF-қB activation. NF-қB inhibitors may reduce the expression of CX3CL1, and modulation of the CX3CL1/CX3CR1 signaling was proposed as a new target for aspirin. We examined the effects of aspirin on CX3CL1 and TNF-α production, as well as CX3CR1 and TNFR1 expression.

**Methods:**

HUVECs isolated after term pregnancies (*N* = 28) were cultured in vitro. Lipopolysaccharide (1 μg/ml) was used as CX3CL1 inducer. HUVECs were exposed to six different concentrations of aspirin (between 1.0 and 6.0 mM) during 7 days. The levels of CX3CL1 and TNF-α in the culture media were measured using ELISA. After termination of the cultures, mean expressions of CX3CR1 and TNFR1 were examined in the immunostained paraffin sections using quantitative immunohistochemistry.

**Results:**

Aspirin significantly (*p* < 0.05) decreased CX3CL1 production, and the mean decrease in CX3CL1 production was inversely proportional to increased (*p* < 0.05) expression of CX3CR1. The combined mean CX3CL1 concentrations, including all time points, equaled 782.18 ± 74.4 pg/ml in aspirin treated HUVECs compared to a total concentration of 2467.53 ± 127.5 pg/ml combined from the respective time points in the controls.

An inhibition of TNF-α production in HUVECs after pretreatment with aspirin was observed. Unlike in the case of CX3CR1 expression, there were no signs of TNFR1 upregulation.

**Conclusions:**

Autoregulation between CX3CL1 and CX3CR1 may explain overexpression of CX3CR1 as the compensatory effect in aspirin-treated HUVECs. Inhibition of CX3CR1 could prevent thrombotic complications in the early period after discontinuation of aspirin.

## Introduction

In 1897, the German chemist Felix Hoffmann was working for Friedrich Bayer and Company and synthesized acetylsalicylic acid (ASA) for the first time in a stable form that was usable for medical applications. In 1899, this compound was launched under the trade name Aspirin by Bayer Company [1]. After many years, aspirin is still one of the most widely used medications worldwide because of its anti-clotting, analgesic, anti-pyretic, and anti-inflammatory properties. The results of many studies provided detailed disclosure of the main mechanisms of aspirin’s action, indicating potential benefits towards the preventive treatment of heart attacks and strokes [2–4]. Aspirin produces irreversible inactivation of cyclooxygenase (COX), an enzyme required for the conversion of arachidonic acid into important mediators known as prostanoids, including thromboxanes, prostacyclin and other prostaglandins. With regard to this property, aspirin differs from most other non-steroidal anti-inflammatory drugs (NSAIDs), which are reversible inhibitors [3]. Aspirin-related inhibition of COX is a consequence of the acetylation of a specific serine moiety (serine 530 and serine 516 of the COX-1 and COX-2 isoforms, respectively) [5]. Although it is a non-selective COX inhibitor, aspirin given in lower doses is approximately 170-fold more potent in inhibiting COX-1 than COX-2 [5]. Other effects of aspirin have also been reported, such as the uncoupling of oxidative phosphorylation in mitochondria and an influence on nuclear factor kappa-light-chain-enhancer of activated B cells (NF-κB) dependent intracellular signaling pathways [6]. This latter effect of aspirin, influencing a local endothelial-related cytokine network, may be linked to delayed progression of atherosclerosis [7, 8].

The chemokine CX3CL1 (fractalkine, neurotactin) is unique in that it exhibits properties of both a chemoattractant (the soluble form of the protein) and an adhesive compound (the cell membrane-anchored form); therefore, CX3CL1 is an important player in the pathogenesis of different inflammatory diseases, including atherosclerotic plaque formation [9]. To date, encoded on human chromosome 16 and possessing three amino-acid residues located between the first two cysteine residues in the molecule, CX3CL1 is the lone member of the CX3C (delta) subfamily of chemokines [10]. The biological activities of CX3CL1 are mediated by its sole receptor CX3CR1 (previously denoted as V28), a Gα_i_ protein-linked seven-transmembrane receptor [11]. CX3CR1 is expressed in endothelial cells, mast cells, monocytes, natural killer (NK) cells, microglial cells, neurons, and subpopulations of T-lymphocytes [12]. Following CX3CR1 stimulation, activation of both CX3CL1-dependent and integrin-dependent migration of cells with augmented adhesion in result of synergistic reactions should be expected. CX3CL1 shows potent chemoattractant properties for NK cells, T cells and monocytes, but not neutrophils. Moreover, CX3CL1 exerts cytotoxic effects on endothelial cells [13]. Endothelial cells express substantial levels of CX3CL1, and considering additional synergistic effects of TNF-α and IFN-γ, almost every stimulus influencing cell homeostasis may induce CX3CL1 secretion [14, 15].

Inhibition of the CX3CL1/CX3CR1 signaling pathway ameliorated the severity of atherosclerosis in animal models [16]. Epidemiological human studies revealed that decreased activity of the CX3CL1/CX3CR1 pathway due to genetic factors may be associated with a lower risk of atherosclerosis as well as a decreased incidence in the episodes of plaque destabilization [17]. CX3CL1 may induce its own expression via the PI3-kinase/PDK1/Akt/NIK/IKK/ nuclear factor kappa beta (NF-қB) signaling pathway [18].

TNF-α also induces the expression of fractalkine and CX3CR1 in the vascular cells, and this induction is mediated by NF-қB activation [19]. The protein encoded in humans by the TNFRSF1A gene is one of the major receptors for TNF-α, and TNFR1(CD120a) can be detected in almost all cell types, including endothelial cells. This receptor can activate the transcription of NF-қB, modulates the inflammatory response and mediates apoptosis [20].

Having proved that specific NF-қB inhibitors markedly reduces the expression of CX3CL1, modulation of the CX3CL1/CX3CR1 signaling pathway was recently proposed as a new target for aspirin [21, 22].

The aim of this preliminary study was to examine the effects of different doses of aspirin on CX3CL1 and TNF-α production, as well as CX3CR1 and TNFR1 expression in human umbilical vein endothelial cells (HUVECs).

## Material and Methods

The study was conducted in compliance with international and local laws regarding human experimentation and was officially approved by the local ethics committee. Written consent was obtained from the women for use of their extraplacental membranes. This study was carried out in accordance with The Code of Ethics of the World Medical Association (Declaration of Helsinki) for experiments involving humans, and the Uniform Requirements for manuscripts submitted to Biomedical Journals have been fulfilled.

HUVECs were isolated from umbilical cords (*N* = 28) obtained from uncomplicated single pregnancies delivered at term by elective cesarean sections. The indications for cesarean section were: high grade myopia in the pregnant woman and breech presentation of the fetus. A more detailed clinical characteristic of the two homogenous groups are given in Table 1. Within a maximum period of 10 min following delivery, fresh umbilical cords were cut off from each placenta with approximately the same length of 15 cm. The outer surface of the cord was cleaned with PBS (Gibco®), and after gently filling the umbilical vein with PBS containing antibiotic/antimycotic (penicillin, streptomycin, and amphotericine B), the cord was clamped on both ends and transported on ice for a short period (approx. 30 min) before HUVEC isolation.

**Table 1:** Clinical characteristics of the two groups studied. The donors of the umbilical cords (*N* = 28) were pregnant women after uncomplicated single pregnancies terminated by elective cesarean section at term

Age of the mothers in full years (range; mean; median)	24–30; 27; 27
Parity (nulliparous/primiparous mothers)	17/11
Gestational age in days pregnancy	270–282; 277; 275
Blood pressure during pregnancy	All records within normal range^a^
Proteinuria during pregnancy	Not present
Liver blood tests (aminotransferases enzymes, AST and ALT levels)	Within normal range^b^
Smoking during pregnancy	None declared active smoking
Diabetes during pregnancy	Not present
Body mass index <21 or >35	None
Mother’s blood (III trimester): hematocrit (Ht), hemoglobin (Hb), red blood cell (RBC) count, mean cell hemoglobin concentration (MCHC)	All within normal range^c^
Other identified risk factors	None
Birth weight in grams (range; mean; median)	3,050–3,980; 3,570; 3400
Sex of newborns (M—male; F—female)	15M + 13 F
Weight of placenta in grams (range; mean; median)	552–825; 655; 649

^a^The normal range of blood pressure was define as systolic pressure between 100 and 140 mmHg, and diastolic pressure between 60 and 90 mmHg

^b^The normal range of values for AST is 5–40 units per liter of serum and the normal range of values for ALT is 7–56 units per liter of serum

^c^Hb levels 10.0–13.5 g/dl; RBC count 3.2–4.4 million/μl; MCHC 319–355 g/L; Ht 31–41 %

### Cell Culture & Determination of CX3CL1 Levels

The method of HUVEC isolation was similar to the procedure described by Crampton et al. [23]. Collagenase treatment was performed, and isolated cells were cultured on plates coated with gelatin. Briefly, fresh, precise cuts on both ends of the cord were performed, and a 21 1/2 G needle with a plastic needle sheath was inserted into the lumen of the vein and then clamped into place with a hemostat. Hank’s balanced salt solution (HBSS; Gibco® Inc., Grand Island, New York, USA) in a 20 cc syringe was attached to the needle, and the vein was flushed twice to eliminate blood residues and the PBS. Next, the 20 cc syringe was disconnected, and a 10 cc syringe was attached to push 10 ml of 0.1 % collagenase into the umbilical vein. After the short flushing phase, the open end of the cord was clamped, and the vein was filled with collagenase until moderate distention occurred. The incubation phase in Ca/Mg free Dulbecco’s phosphate-buffered saline (DPBS; Gibco®) at 37 °C for 20 min was accompanied by gently massaging of the cord. After incubation, all effluent was collected in a conical tube containing fetal bovine serum (FBS) and centrifuged for 5 min at 1,200 grm at room temperature. The supernatant was aspirated, and the cell pellets were resuspended in standard CS-C medium (Sigma, St. Louis, MO, USA) supplemented with endothelial cell growth factor and endothelial cell attachment factor. HUVECs were seeded at a density of 1 × 10^5^ cells/well in gelatinized 24-well culture plate inserts. A total of 56 plates were seeded (2 plates per 1 umbilical cord) with HUVECs. The yield and viability of the isolated HUVECs were assessed by the Trypan blue exclusion assay. Twenty-four hours after plating, lipopolysaccharide (LPS, 1 μg/ml) was added to the culture media to induce secretion of CX3CL1. Then, the HUVECs were continuously exposed to aspirin (Sigma) administered in 6 different concentrations (1.0, 2.0, 3.0, 4.0, 5.0, and 6.0 mM) in respective groups (designated as groups I to VI, and the control, aspirin free group was designated as group VII) during the 7 day culture period in normoxia at 37 °C. A colorimetric assay utilizing 3-(4.5-dimethylthiazol-2-yl)-2.5-diphenyltetrazolium bromide (MTT; Sigma) was used to assess HUVEC viability before and after exposure to the given doses of aspirin (the time points 24, 48, 72, and 144 h.

The levels of CX3CL1 in the culture media supernatants were measured at 24, 48, 72, and 144 h time points using a RayBio® Human Fractalkine ELISA Kit (RayBiotech, Inc., USA). According to the manufacturer’s information, the minimum detectable dose of CX3CL1 for this test is typically less than 300 pg/ml, and cross reactivity was not observed with any of the cytokines tested including human angiogenin, BDNF, BLC, ENA-78, FGF-4, IL-1α, IL-1β, IL-2, IL-3, IL-4, IL-5, IL-7, IL-8, IL-9, IL-10, IL-11, IL-12 p70, IL-12 p40, IL-13, IL-15, IL-309, IP-10, G-CSF, GM-CSF, IFN-γ, leptin, MCP-1, MCP-2, MCP-3, MDC, MIP-1α, MIP-1β, MIP-1δ, PARC, PDGF, RANTES, SCF, TARC, TGF-β, TIMP-1, TIMP-2, TNF-α, TNF-β, TPO, and VEGF.

### Determination of the TNF-α Concentration in the Culture Media

Additionally, measurements of the TNF-α level in the culture supernatants were performed among all studied HUVEC groups by ELISA with the same timing adopted for the CX3CL1 concentration assessment. A commercially available kit was used (ELH-TNFalpha-001, RayBio Human TNF-alpha ELISA Kit, RayBiotech, Inc., USA) according to the manufacturer’s instructions. As assured by the producer, the minimum detectable dose of TNF-α was typically less than 10 pg/ml.

### Immunohistochemistry and Mean Expression of CX3CR1

To visualize CX3CR1 by immunohistochemistry, the cultures were terminated, formalin fixed and paraffin embedded. Both initial (E_i_; after LPS exposure but not exposed to aspirin) and final (E_f_; after 7 days of culture) expressions of the fractalkine receptor CX3CR1 were compared within and between studied groups. Standard immunohistochemical procedures were applied. Rabbit polyclonal antibody IgG to CX3CR1 (ab8020; Abcam Inc., USA; concentration of 10 μg/ml) was used as the primary antibody and a goat anti-rabbit IgG biotinylated antibody was used as the secondary antibody (ab64256; Abcam; 0.5 % v/v). Visual detection of the primary anti-receptor antibodies was performed using the StreptABComplex/HRP Duet (Dako Cytomation, Glostrup, Denmark) following the procedure recommended by the manufacturer, with 3,3′-diaminobenzidine serving as a chromogen. The negative controls for immunostainings were set up by replacement of the polyclonal primary antibody with normal rabbit pre-immune IgG diluted with phosphate buffered saline containing 3 % bovine serum albumin at the same protein concentration as that used for the primary antibody.

Thereafter, quantitative immunohistochemistry based on morphometric software (Quantimet 500C+, Leica, UK) was applied for CX3CR1 receptor identification in paraffin-embedded 5 μm sections of the HUVECs cultures under light microscopy. For comparison and validation, all these morphometric operations were carried out twice by two independent observers, and the average results were recorded. The intensity of immunostaining was evaluated using a mean color saturation parameter and thresholds in grey-level histograms. The expression of CX3CR1 corresponded to the total immunostained calibrated area of examined sections, whereas color saturation comprised segmentation-separation criteria for objects. A single analyzed image area was 138,692 μm^2^ (magnification x 200). For each umbilical cord collected as a source of the HUVECs culture, twelve visual fields were analyzed. In all studied groups, 336 visual fields were analyzed (168 visual fields per each time point marked E_i_ and E_f_ as previously stated). Thus, 24 visual fields were analyzed within each group I-VII (12 at time point E_i_ and 12 at E_f_). To assure optimal accuracy of measurements, the following factors have been controlled for or monitored: illumination, power supply, warming up, shading correction, averaging of image intake, hue, luminance, and relation of illumination to quantification of area percentage of positively staining structures. A more detailed description of these morphometric procedures is given elsewhere [24, 25]. Morphometric results comprising 90 % confidence intervals were reported as the mean percentage values ± SEM.

### Immunohistochemistry and Mean Expression of TNF-α Receptor Type 1 (TNFR1)

To identify the TNF-α receptor type 1 (TNFR1, TNFRSF1A, CD120a), an immunohistochemical staining procedure was utilized with a goat anti-human polyclonal antibody IgG to TNF R1/TNFRSF1A (AF225; R&D Systems, Inc., USA; concentration of 10 μg/ml) as the primary antibody, and an Anti-Goat HRP-AEC Cell & Tissue Staining Kit (CTS009; R&D) was used to obtain formation of the Avidin-Biotin Complex (ABC) followed by visualization based on enzymatic conversion of a chromogenic substrate 3-amino-9-ethylcarbazole (AEC) into colored red precipitate by horseradish peroxidase (HRP) at the sites of antigen localization.

Identification of HUVECs immunostained for TNFR1 in paraffin-embedded 5 μm sections using quantitative immunohistochemistry was performed in an identical manner as described for CX3CR1, which means that both initial (E_i_; after LPS exposure but not exposed to aspirin) and final (E_f_; after 7 days of culture) expressions of TNFR1 were compared.

### Detection and Quantification of NF-қB in the HUVECs Lysates

To investigate comparatively the influence of different doses of aspirin on NF-қB signaling, the sandwich ELISA assay method was applied. Following the cultures’ termination at Day 7, the cell lysates were prepared for detection and quantification of the level of NF-қB/p65 protein using 96-well plates and a microplate reader. In each of the groups, the cell lysates were prepared from an equal number of HUVECs (1 × 10^4^ cells/100 μl) by addition of ice-cold phosphoprotein lysis buffer (30 μl for each well) containing 4 mM sodium pyrophosphate, 50 mM HEPES, 100 mM NaCl, 10 mM EDTA, 10 mM NaF, 2 mM NaVO4 with 1 mM PMSF, 10 % Triton X-100, 5 μg/ml leupeptin and 5 μg/ml aprotinin. The Invitrogen NF-қB/p65 [total] ELISA Kit [KHO0371; Novex®] was used, and the level of NF-қB/p65 protein was assessed independent of its phosphorylation state. The analytical sensitivity of this assay was <50 pg/ml for NF-қB/p65. The p50 (NF-қB1)/p65 (RelA) heterodimer assessed by the mentioned ELISA test is the most abundant form of NF-қB [26].

### Statistical Analysis

The results were expressed as the mean ± SEM or mean percentage value ± SEM and were compared by analysis of variance. Post-hoc Student’s t-tests or post-hoc Mann-Whitney’s *U*-test were applied. Differences in the mean concentration of CX3CL1, TNF-α and NF-қB among groups I to VII as well as the differences between CX3CR1 expression were deemed statistically significant if *p* < 0.05.

## Results

**Fig. 1:**
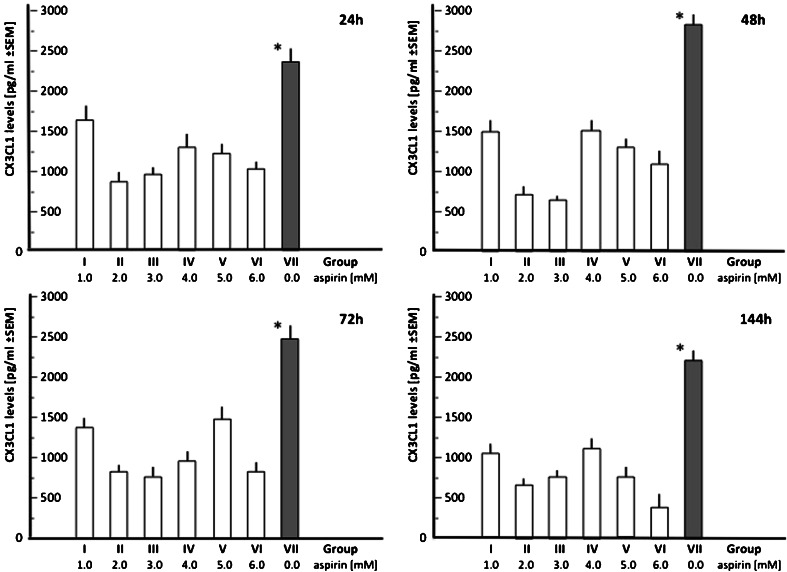
Relationships between aspirin dose and the mean CX3CL1 levels in the culture media at the consecutive time points

**Fig. 2:**
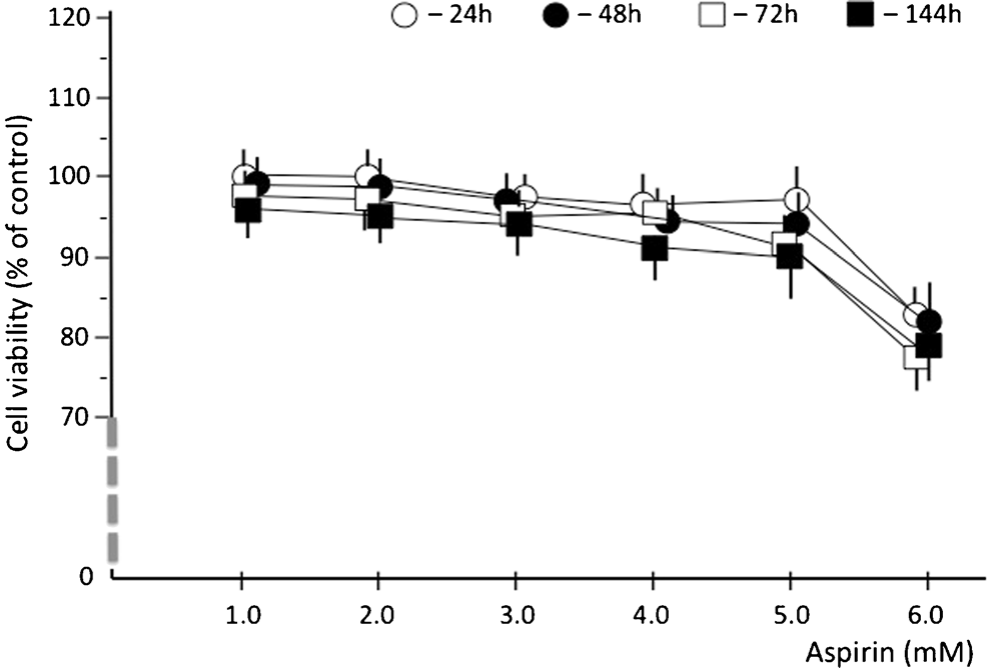
Cell viability assay of HUVECs exposured to different doses of aspirin over time (at a given time points)

Aspirin treatment produced a significant (*p* < 0.05) decrease in the mean concentration of CX3CL1 in the HUVECs culture supernatant, especially when administered at doses of 2.0 and 3.0 mM (Fig. 1, Tables 2 and 3). This curtailment of CX3CL1 production persisted throughout the whole period of observation (time points 24, 48, 72, and 144 h). The combined mean CX3CL1 concentration for group II and group III, including all time points, equaled 782.18 ± 74.4 pg/ml compared to a total concentration of 2467.53 ± 127.5 pg/ml combined from the respective time points in group VII (control). Analysis of the cell viability in the HUVEC cultures revealed that the highest tested dose of aspirin (6.0 mM) resulted in a significant increase in cytotoxicity (Fig. 2). This effect was observed at the beginning (24 h after aspirin administration), and the mean cell viability still lingered below the mean values obtained in the controls (*p* < 0.05) at the respective time points.

**Table 2:** CX3CLI levels (pg/ml) in the HUVECs cultures measured ath the consecutive time points

Time point	Group (aspirin dose [mM]						
	I (1.0)	II (2.0)	III (3.0)	IV (4.0)	V (5.0)	VI (6.0)	VII (0.0; control)
Time after induction by LPS							
24 h							
Mean	1670.3 ± 166	880.8 ± 97	984.4 ± 78	1302.5 ± 141	1247.3 ± 103	1036.6 ± 79	2352.7 ± 140
Median	1692	819	801	1234	1266	890	2401
Range	1020–2004	473–1354	422–1251	719–1603	705–1394	368–1310	1872–3335
48 h							
Mean	1498.1 ± 111	741.0 ± 89	678.7 ± 40	1504.2 ± 104	1312.5 ± 92	1036.6 ± 79	2833.9 ± 105
Median	1524	762	670	1544	1272	1141	2912
Range	913–1743	407–1175	379–1075	772–1893	665–1733	368–1362	2210–3608
72 h							
Mean	1352.6 ± 107	813.8 ± 47	754.5 ± 104	942.3 ± 102	1470.3 ± 151	812.8 ± 99	2469.1 ± 156
Median	1295	773	701	959	1358	639	2370
Range	614–1752	481–1175	493–1032	518–1687	576–1769	330–1070	1903–2952
144 h							
Mean	1058.3 ± 98	652.8 ± 69	751.4 ± 71	1099.8 ± 110	752.3 ± 112	372.6 ± 160	2214.4 ± 109
Median	1008	680	713	982	844	404	2115
Range	450–1491	414–960	428–973	350–1191	409–1303	305–487	1720–2641

Mean ± SEM, median (rounded to the nearest whole number), and 95 % confidence interval (95 % CI) are shown

**Table 3:** TNF-α levels (pg/ml) in the HUVECs cultures measured at the consecutive time points

Time point	Group (aspirin dose [mM]						
	I (1.0)	II (2.0)	III (3.0)	IV (4.0)	V (5.0)	VI (6.0)	VII (0.0; control)
Time after induction by LPS							
24 h							
Mean	940.1 ± 89	769.0 ± 119	436.3 ± 73	309.5 ± 74	314.5 ± 59	250.4 ± 45	998.9 ± 117
Median	955	748	449	328	303	264	1046
Range	510–1597	400–1412	293–652	202–651	221–543	134–478	679–2005
48 h							
Mean	914.7 ± 104	725.4 ± 88	361.2 ± 77	316.8 ± 58	252.2 ± 60	227.6 ± 74	1104.7 ± 106
Median	900	732	377	325	249	238	1046
Range	587–1423	366–1377	187–769	214–601	110–433	125–440	679–1824
72 h							
Mean	698.3 ± 103	438.1 ± 104	269.4 ± 58	206.3 ± 61	187.9 ± 44	270.3 ± 76	11021.2 ± 118
Median	703	399	239	201	214	262	1037
Range	345–1098	216–749	173–418	111–429	95–356	154–560	603–1692
144 h							
Mean	561.9 ± 92	303.4 ± 90	212.8 ± 45	135.7 ± 40	250.4 ± 57	266.8 ± 61	938.2 ± 92
Median	505	307	194	148	260	231	904
Range	269–978	168–515	96–442	79–261	144–408	100–513	572–1706

Mean ± SEM, median (rounded to the nearest whole number), and 95 % confidence interval (95 % CI) are shown

**Fig. 3:**
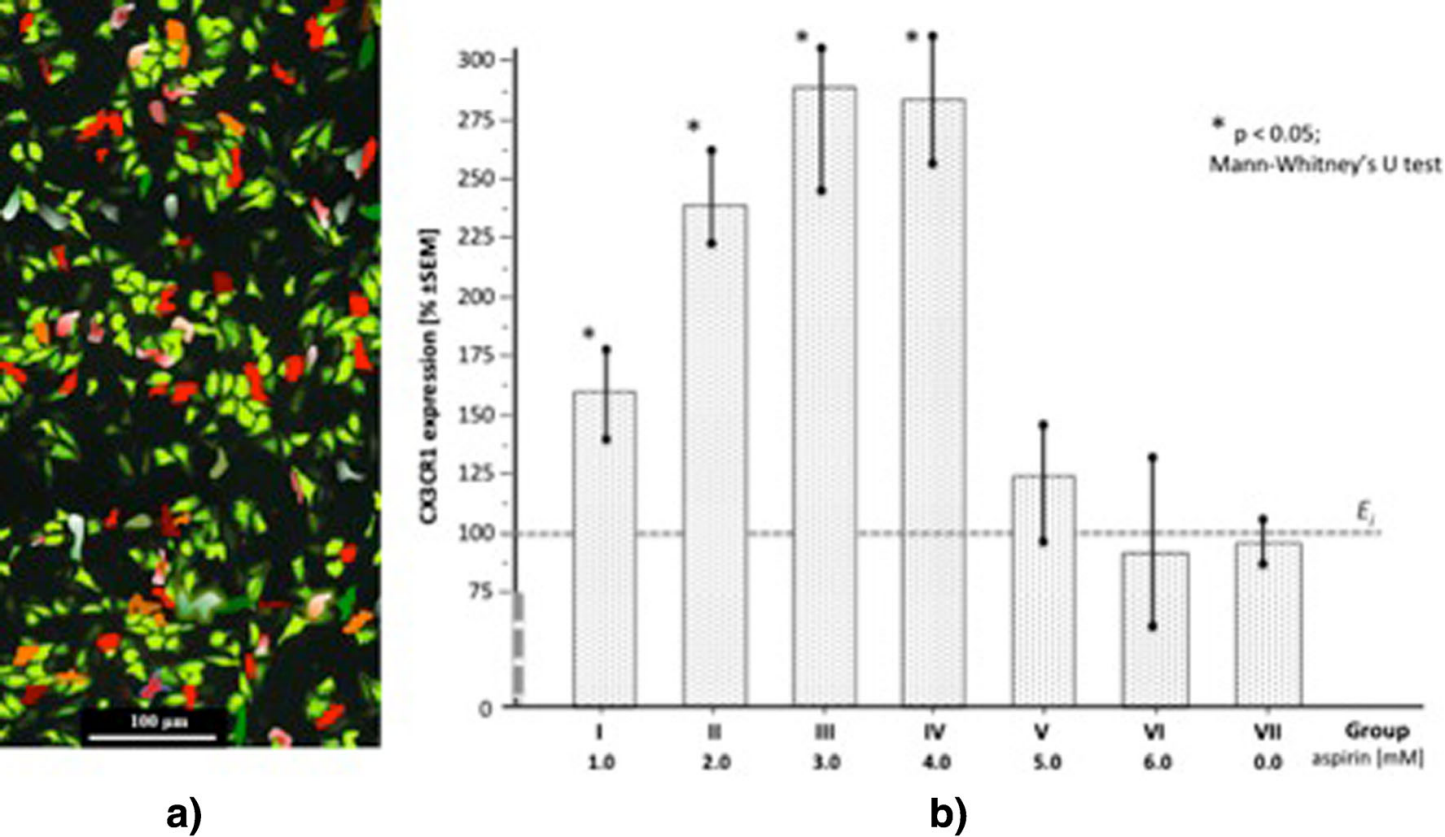
**a.** Immunohistochemical visualization of the receptor CX3CR1 in HUVECs culture (the tones of orange-red-brown color; the image captured through optical microscope was digitally transformed for morphometric purposes); **b.** Mean final expressions (E_f_) of CX3CR1 in HUVECs cultures exposured to different doses of aspirin (groups I to VI), including control group VII. The mean initial value of CX3CR1 expression (E_i_) was taken as 100 % for each group. Twenty four visual fields were analysed within each group I–VII (12 at time point E_i_ and 12 at E_f_, respectively)

The overall quality of CX3CR1 immunostaining in cultured HUVECs was sufficient for the application of standardized quantitative immunohistochemistry to investigate expression of this receptor (Fig. 3a). The mean decrease in CX3CL1 production was inversely proportional to the significantly increased (*p* < 0.05) mean final expression (E_f_) of CX3CR1 (% of the initial expression [E_i_], ± SEM) in HUVECs for aspirin concentrations 1.0, 2.0, 3.0, and 4.0 mM, amounting to 159.71 ± 37.7, 237.98 ± 40.4, 288.6 ± 51.3, and 282.9 ± 47.6 pg/ml, respectively (Fig. 3b). The change between the final and the initial CX3CR1 expression was not significant in the control group (group VII).

**Fig. 4:**
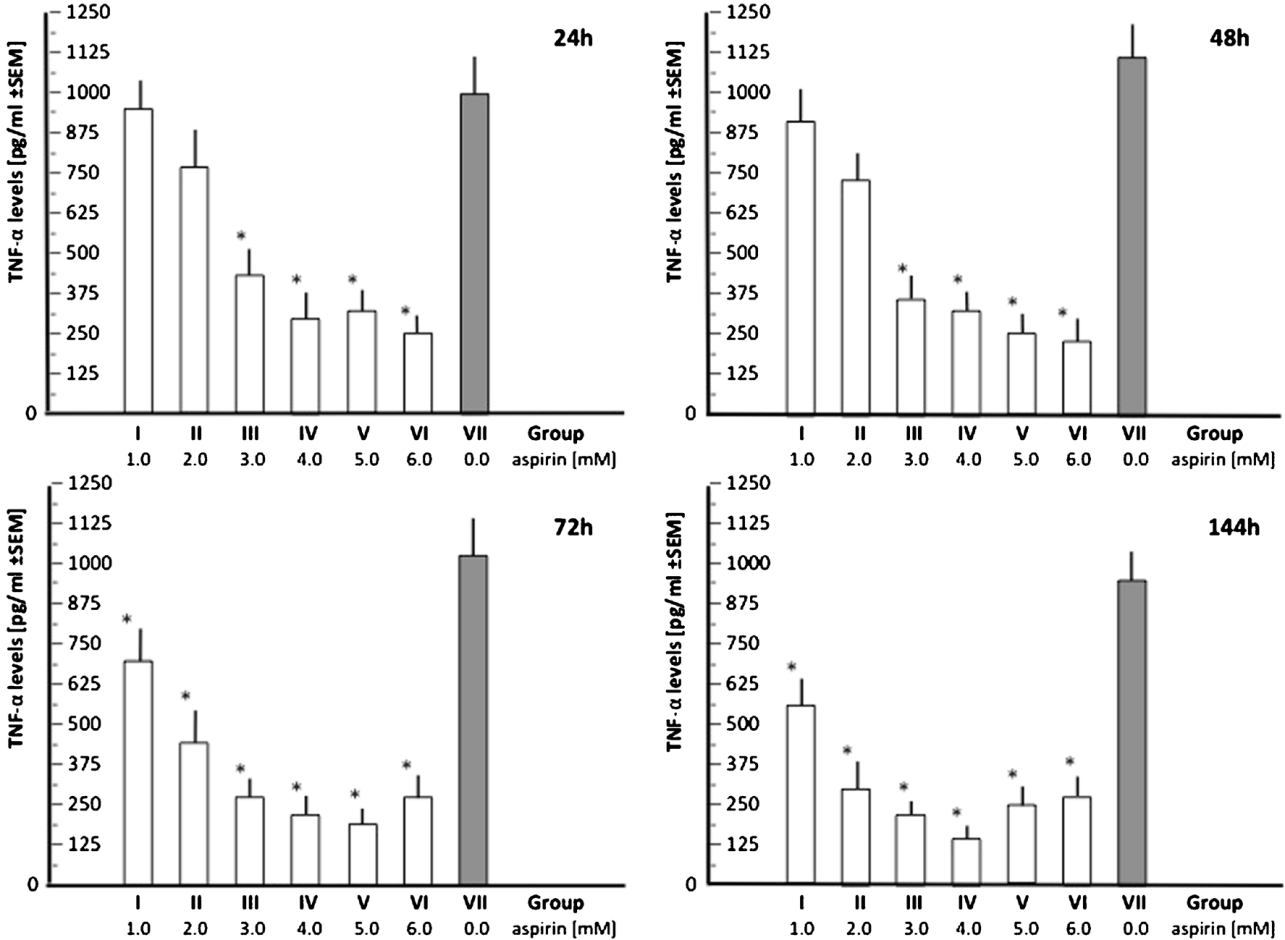
Relationships between aspirin dose and the mean TNF-α levels in the culture media at the consecutive time points

The relationships between aspirin dose and the mean TNF-α level in the culture media are shown in Fig. 4. A significant inhibition of TNF-α production in HUVECs after pretreatment with aspirin was observed throughout the whole period of observation (time points 24, 48, 72, and 144 h) for doses 3.0, 4.0, 5.0, and 6.0 mM. As in the case of CX3CL1, analysis of the cell viability (Fig. 2) should raise suspicion that the results for the highest doses of aspirin are influenced by increased cytotoxicity in the cultured HUVECs. The mean TNF-α level assessed together for groups III, IV, and V, comprising all time points, equaled 271.1 ± 58.8 compared to 1015.75 ± 108.3 pg/ml summed together from the same time points in group VII (control).

**Fig. 5:**
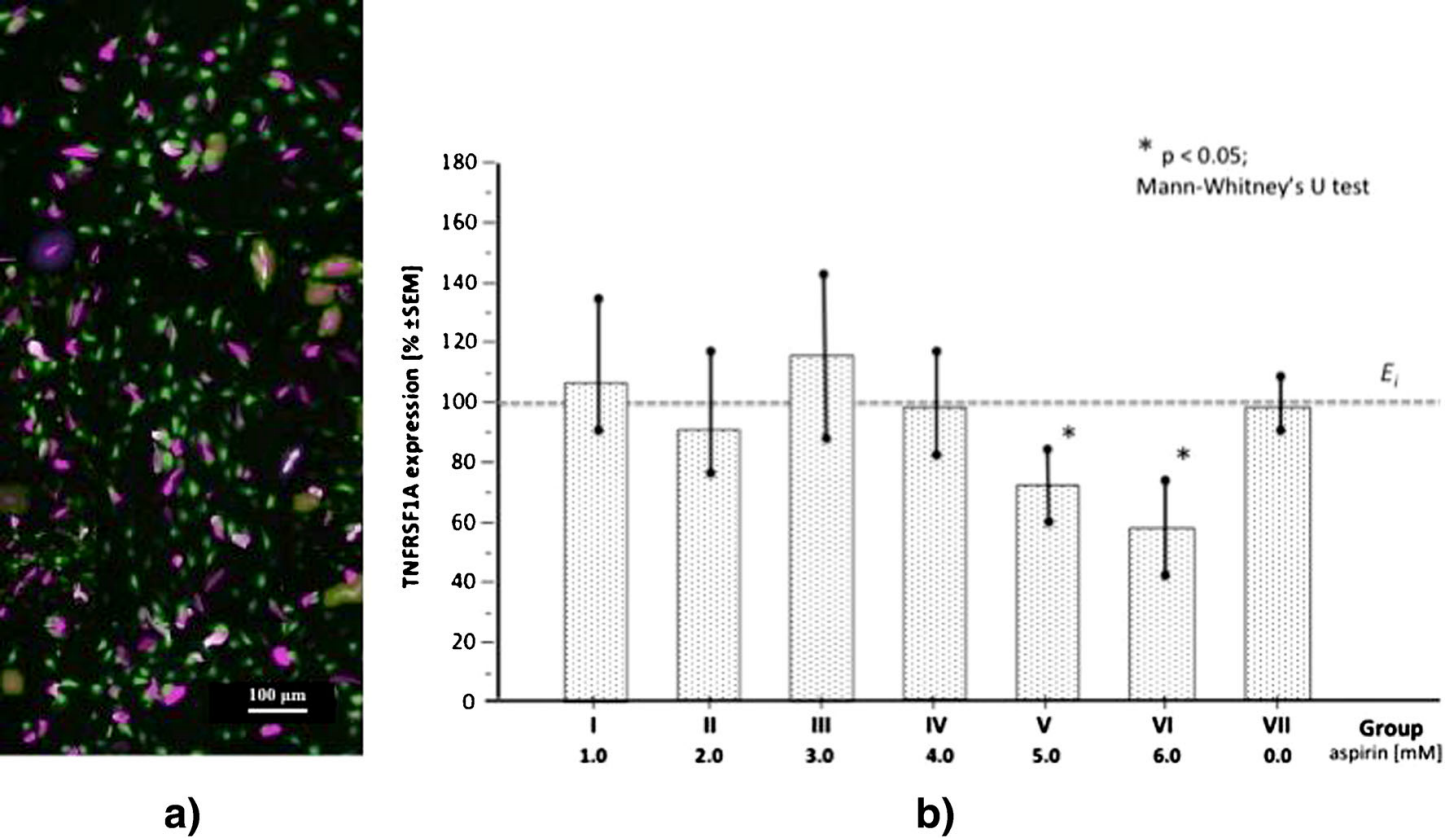
**a.** Immunohistochemical visualization of the receptor TNFR1 (TNFRSF1A) in HUVECs culture (the tones of pink-purple color; the image captured through optical microscope was digitally transformed for morphometric purposes); **b.** Mean final expressions (E_f_) of TNFRSF1A in HUVECs cultures exposured to different doses of aspirin (groups I to VI), including control group VII. The mean initial value of TNFRSF1A expression (E_i_) was taken as 100 % for each group. Twenty four visual fields were analysed within each group I–VII (12 at time point E_i_ and 12 at E_f_, respectively)

The methodological correctness of the immunostaining procedure was confirmed, and visualization of the TNFR1 was adequate for its expression assessment using quantitative immunohistochemistry (Fig. 5a). Unlike in the case of CX3CR1 expression, there were no signs indicating upregulation of TNFR1. The mean expression of the receptor after 7 days of culture (E_f_) did not significantly differ from the starting point before aspirin treatment (E_i_) but were significantly lower (*p* < 0.05) in groups V and VI (Fig. 5b). However, the results in the two latter groups may reflect augmented cytotoxicity due to higher doses of aspirin (5.0 and 6.0 mM, respectively). Nonetheless, despite the aspirin-related decreased production of TNF-α in cultured HUVECs, the mean expression of its main receptor TNFR1(TNFRSF1A) remained unchanged or was slightly (*p* > 0.05) reduced (which should be interpreted with caution, considering cytotoxicity) compared to the initial values (E_i_) obtained without pretreatment with aspirin. The difference between the final and initial expression of CX3CR1 in the control (aspirin-free) group VII did not reach statistical significance (*p* > 0.05).

**Fig. 6:**
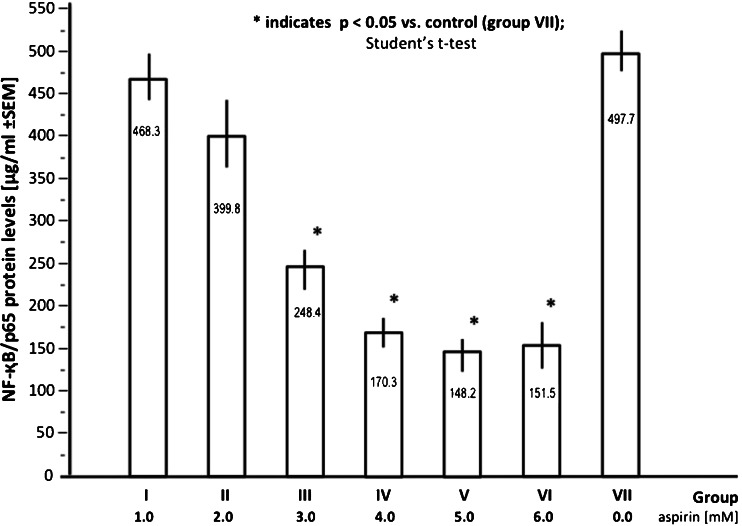
Relationship between aspirin dose and the mean NF-қB (NF-қB/p65 protein heterodimer) concentrations in HUVECs cell lysates. The cultures were subjected to LPS pretreatment (1 μg/ml)

The levels of NF-қB/p65 reflected augmented production of these proteins in HUVECs under the influence of LPS, a known NF-қB inducer, administered initially (24 h after the cells were plated). Examination of the HUVEC cell lysates revealed a negative correlation between the administered dose of aspirin and the NF-қB/p65 protein level (Fig. 6). In groups III, IV, V, and VI (aspirin doses 3.0, 4.0, 5.0, and 6.0 mM, respectively), NF-қB (NF-қB/p65 protein heterodimer) concentrations were significantly (*p* < 0.05) lower (approximately 2-fold to 3.4-fold) compared to the control group.

## Discussion

Altered endothelial function due to microinjury or altered metabolic activity may produce leukocyte adhesion and leukocyte transendothelial migration (diapedesis), which takes part in the pathomechanism of atheroscelerotic plaque formation [27]. Among many other players, cytokine TNF-α and chemokine CX3CL1, with unique biochemical properties, are undoubtedly involved in the destructive process that leads to an increased propensity for coagulation and thrombosis within the endothelial barrier [16]. Significant changes in the activity of the genes that code for TNF-α and CX3CL1 have been reported at the site of atherosclerosis [8, 13]. This study revealed that aspirin administered within the therapeutic dose range produces a dose-dependent reduction in the level of both proinflammatory compounds in cultured HUVECs under normoxia. According to the observed inhibition of NF-κB production, this effect is mediated through a COX-independent pathway as described by others [28, 29].

As reported in this study, the substantive differences between TNFRSF1A and CX3CR1 expressions are clearly visible and deserve in-depth discussion of the mechanisms involved, including the nature of interactions between TNF-α and CX3CL1 under conditions of NF-κB inhibition. In other words, more studies are needed to explain why aspirin treatment upregulates CX3CR1 in HUVECs while leaving TNFRSF1A expression unchanged.

Some authors emphasized the importance of an autocrine effect of CX3CL1-induced CX3CR1 expression on the basis of their studies performed in aortic smooth muscle cells and neurons [18, 30]. Because HUVECs, like most other endothelial cells, express CX3CL1 and CX3CR1, existence of the analogy is justified [31]. Such co-expression may produce an acute induction of CX3CL1 by a wide range of proinflammatory factors, including TNF-α, LPS and hyperlipidemia, and may lead to vascular injury and atherosclerosis [16, 31–33]. For example, the results pertaining to murine and human platelets in hyperlipidemic environment suggest a significant role of the CX3CL1/CX3CR1 signaling in platelet accumulation and monocyte recruitment at sites of arterial damage [33].

The proinflammatory, pleiotropic, and homotrimeric soluble cytokine TNF-α is implicated in some metabolic disorders with an inflammatory background including, but not limited to, atherosclerosis [34]. TNF-α leads to the activation of caspases and the two transcription factors activation protein-1 (AP-1) and NF-κB. Acting through its type 1 receptor TNFR1(TNFRSF1A), TNF-α typically initiates transcriptional responses through the rapid activation of NF-κB (within 10–15 min) and/or AP-1 (within 15–30 min) [35]. Co-stimulation of cells with TNF-α, LPS, and interferon gamma (IFN-γ) may lead to synergistic super induction of the chemokine CX3CL1 in endothelial cells [14].

TNF-α induces the expression of CX3CL1 and CX3CR1 in a time-dependent manner through at least two signaling pathways that converge at the point of IKK activation [18, 36]. The IKK complex is the core element of the NF-κB cascade. The trimeric structure of TNF-α corresponds to the respective trimerization of the TNFR1. After ligand binding to TNFR1, several signaling proteins are recruited to the cytoplasmic domain of TNFR1, including the adapter protein TRADD (TNFR1-associated death domain). Binding of TRADD and FADD (fas-associated death domain) to TNFR1 leads to the recruitment, oligomerization, and activation of Caspase 8. Activation of this caspase initiates a proteolytic cascade and ultimately induces apoptosis. TRADD–TRAF2 (TNF receptor associated factor 2) complex formation is the other way to induce TNF-α/TNFR1 signaling activity that leads to activation of NIK, IKK, and NF-κB [37].

TNF-α dependent activation of NF-κB, CX3CL1 production, as well as CX3CR1 expression may be significantly influenced by the changes in SODD (silencer of death domains), a widely expressed 60 kDa protein associated with the DD of TNFR1 [38]. SODD is probably involved in a general mechanism for preventing spontaneous signaling by DD-containing receptors. Overexpression of SODD associated with the DD of TNFR1 suppresses biological activities of TNF-α, indicating that SODD plays a role as a negative regulatory protein for TNF-α/TNFR1 signaling [39]. A recent report demonstrated that expression of SODD significantly declined following aspirin exposure [38]. This observation is consistent with the well-documented pro-apoptotic activity of aspirin [40].

Our results may indicate that the effect of the decreased TNF-α that accompanies aspirin treatment may be additionally augmented by the fact that significant modulation of the TNF-α/TNFR1 signaling pathway causes a decrease in NF-κB production by HUVECs, which may coexist with non-changed expression of the TNFR1. The mean level of CX3CL1 also declines corresponding to the decreased concentration of TNF-α (a weaker stimulator of CX3CL1 production). In contrast, upregulation of CX3CR1 may be a logical consequence of autoregulation of this receptor by the mechanism which efficiently utilizes signaling via heterotrimeric G_i_ proteins, PI-3 kinase, PDK1, Akt, NIK, IKK and NF-κB, independent of SODD expression. Thus, a significant reduction in the level of NF-κB may induce a negative autoregulatory loop with an observed increase in the mean CX3CR1 expression. Further studies are needed to reveal the relationship between such overexpression of CX3CR1 and the reported clinical observation from patients with indications for thrombolytic therapy that the risk of cardiovascular thrombotic events increases shortly after discontinuation of acetylsalicylic acid [41–43].

Interpretation of our results pertains only to normoxic primary HUVECs. To obtain maximum homogeneity of the cultures and optimal reproducibility of the results, endothelial cell lines should be used in future studies. Realizing that pre-existing infection, hypoxic conditions and/or the use of cancer cell cultures in experimental models can produce different results is also important [44, 45]. The activation of signaling pathways involved in CX3CL1/CX3CR1 regulation in response to oxygen deprivation (e.g., pro-angiogenic) or related to cancerous mutations have been studied by many researchers. Changes in the cytokine network including TNF-α and CX3CL1 production and their respective receptor expression were striking [46, 47].

Moreover, the mean expression of the other receptor belonging to the TNF receptor superfamily, TNFR2 has not been studied here. Confirmed in endothelial cells, the expression profiles, ligand affinities, cytoplasmic tail structure, and downstream signaling pathway activation of TNFR2 differ from that documented for TNFR1 [20]. This receptor deserves our further attention because it is clearly evident from analyses of TNFR1 knock-out mice that TNFR2 stimulation alone is sufficient to activate most of the signaling pathways mediated via TNFR1 [20].

In conclusion, using aspirin concentrations within the therapeutic range in cultured HUVECs, we observed that the TNF-α level decreased without significant changes in the mean TNFR1 expression, and inhibited production of CX3CL1 was accompanied by up regulation of CX3CR1. Because LPS-induced expression of CX3CL1 in many endothelial cells involves activation of the transcription factor NF-kB, the mechanism of the anti-aggregatory action of aspirin may be secondary to the inhibition of NF-kB, which then reduces the local concentrations of CX3CL1 [21, 29]. Existence of an autoregulatory mechanism between CX3CL1 and CX3CR1 may explain the increased expression of CX3CR1 as the compensatory effect in aspirin-treated HUVECs. Pharmacological inhibition of the chemokine receptor CX3CR1 should be considered as a preventative measure for thrombotic complications in the early period after discontinuation of aspirin therapy.
